# EsPRit: ethics committee proposals for Long Term Medical Data Registries in rapidly evolving research fields - a future-proof best practice approach

**DOI:** 10.1186/s12911-017-0539-9

**Published:** 2017-10-18

**Authors:** S. Oberbichler, W. O. Hackl, A. Hörbst

**Affiliations:** 1 eHealth Research and Innovation Unit, UMIT – University for Health Sciences, Medical Informatics and Technology, Hall in Tirol, Austria; 2 Institute of Biomedical Informatics, UMIT – University for Health Sciences, Medical Informatics and Technology, Hall in Tirol, Austria

**Keywords:** Clinical research, Long-term data collection, Medical data registry, Ethics committee proposal

## Abstract

**Background:**

Long-term data collection is a challenging task in the domain of medical research. Many effects in medicine require long periods of time to become traceable e.g. the development of secondary malignancies based on a given radiotherapeutic treatment of the primary disease. Nevertheless, long-term studies often suffer from an initial lack of available information, thus disallowing a standardized approach for their approval by the ethics committee. This is due to several factors, such as the lack of existing case report forms or an explorative research approach in which data elements may change over time.

In connection with current medical research and the ongoing digitalization in medicine, Long Term Medical Data Registries (MDR-LT) have become an important means of collecting and analyzing study data. As with any clinical study, ethical aspects must be taken into account when setting up such registries.

This work addresses the problem of creating a valid, high-quality ethics committee proposal for medical registries by suggesting groups of tasks (building blocks), information sources and appropriate methods for collecting and analyzing the information, as well as a process model to compile an ethics committee proposal (EsPRit).

**Methods:**

To derive the building blocks and associated methods software and requirements engineering approaches were utilized. Furthermore, a process-oriented approach was chosen, as information required in the creating process of ethics committee proposals remain unknown in the beginning of planning an MDR-LT. Here, we derived the needed steps from medical product certification. This was done as the medical product certification itself also communicates a process-oriented approach rather than merely focusing on content. A proposal was created for validation and inspection of applicability by using the proposed building blocks. The proposed best practice was tested and refined within SEMPER (Secondary Malignoma - Prospective Evaluation of the Radiotherapeutics dose distribution as the cause for induction) as a case study.

**Results:**

The proposed building blocks cover the topics of “Context Analysis”, “Requirements Analysis”, “Requirements Validation”, “Electronic Case Report (eCRF) Design” and “Overall Concept Creation”. Additional methods are attached with regards to each topic. The goals of each block can be met by applying those methods. The proposed methods are proven methods as applied in e.g. existing Medical Data Registry projects, as well as in software or requirements engineering.

**Conclusion:**

Several building blocks and attached methods could be identified in the creation of a generic ethics committee proposal. Hence, an Ethics Committee can make informed decisions on the suggested study via said blocks, using the suggested methods such as “Defining Clinical Questions” within the Context Analysis. The study creators have to confirm that they adhere to the proposed procedure within the ethic proposal statement. Additional existing Medical Data Registry projects can be compared to EsPRit for conformity to the proposed procedure. This allows for the identification of gaps, which can lead to amendments requested by the ethics committee.

## Background

Ongoing developments regarding clinical treatment, diagnoses, technological possibilities in medicine and a constant increase in medical knowledge have led to more diversity in the medical field as ever experienced before. This freedom bears great potential regarding patient treatment but also adds to the complexity. Furthermore, there is an increase in the number of potential dependencies, interrelations and unintended consequences, raising more questions than ever. Along with the current paradigm of evidence-based medicine, many of these questions cannot be answered by short term studies but require long-term investigations.

One possibility for doing that is looking back and reusing available patient data for retrospective analyses. Another, forward-facing approach is to collect a set of defined data elements prospectively in so-called Medical Data Registries (MDR). Of course, combinations of these two diametric paradigms are possible, e.g. to reuse existing clinical data to enrich the prospective MDRs. However, apart from the basic organizational challenge to maintain a complete follow-up of all observed subjects over time, in addition to technological challenges (e.g. changing software requirements), several other challenges for long-term medical data registries (MDR-LT) exist.

These challenges include, for example, changes in treatment guidelines and standards, improved surgical methods, new therapies, novel or phased-out medications, improved or novel radio-diagnostic or laboratory examination methods, etc.

Furthermore, new or future knowledge leading to further insights in the studied field may require additional data to be included in the registry. The available data itself can change considerably in volume, form, representation or granularity over such long periods of record (e.g. changed documentation and coding standards, newly available, personalized omics data). Even the initial research question may change over time and new, interesting aspects may arise based on medical progress, on the already gathered knowledge in an existing registry or based on novel analyzing techniques.

All these challenges and especially possible future changes should be taken into account when planning a future-proof MDR-LT [1]. Versioning of entered data sets has to be provided in order to ensure traceability [2–4]. On the other hand, according to common legal or regulatory aspects and following the Declaration of Helsinki [5], approval of a responsible ethics committee is needed for such registries for the long-term collection, integration and analysis of personal medical data.

The World Medical Association (WMA) formulated the Declaration of Helsinki. The first version dates back to 1964 but was revised several times (current is seventh revision form 2003). The Declaration contains several statements outlining the need of ethical aspects in medical research, as well as the steps to guarantee this. It is stated (Article 11) that “*… Medical research involving human subjects must conform to generally accepted scientific principles, be based on a thorough knowledge of the scientific literature, other relevant sources of information, and on adequate laboratory and, where appropriate, animal experimentation.*” Adhering to these principles ensures coverage of ethical aspects within research in the medical domain. Most ethics committees respect the declaration and incorporate these principles in their deciding processes on whether a study meets ethical. In general, each research project involving humans or animals must undergo an examination determining whether ethical considerations have been respected. It is for that reason that institutions involved in medical research have ethics committees responsible for analyses of requested projects; this process is based on a submitted ethics committee proposal. A recent study in Finland outlines that adherence to rules of good medical research as specified in the international accepted quality standard Good Clinical Practice (GCP) [6] is the major critical component taken into account for an informed decision by the committees [7].

Since, depending on the applicable laws in most countries, approval decisions of said committees must be given in advance. This may lead to the dilemma that a committee is requested to make a decision without knowing how a MDR-LT will look like, what data it will store and how the data will be analyzed in the future.

### Objective

Given this unique situation, it is exceedingly difficult to draft a MDR-LT ethics proposal in a manner that it guarantees its approval while accounting for the needs and dynamics of such a system. Therefore, an approach is needed which allows necessary and reasonable future changes of such a registry (e.g. of collected data), while still guaranteeing that the MDR-LT adheres to strong ethical standards in medical research and meets all requirements as defined by an ethics committee.

The objective of our work is to present a best practice approach to overcome this dilemma: The EsPRit (EthicS Proposal RegIsTry) approach.

EsPRit defines generic building blocks for a proposal including appropriate methods to parameterize these building blocks and subsequently follows a general guideline compiling ethics proposals for MDR-LT projects. EsPRit can be used in two ways: to create an ethics proposal in the planning phase of the MDR-LT project (without the need to know all the details of the desired registry project in advance); to adapt such a proposal during the lifetime of the MDR-LT whenever substantial aspects (e.g. the clinical questions) are altered by applying the attached methods.

EsPRit is based on a thorough literature review and uses components of the Systematic Planning of Intelligent Reuse of Integrated Clinical Routine Data (SPIRIT) framework for the systematic planning of intelligent reuse of integrated clinical routine data [8]. The concept for EsPRit was designed in a radiotherapeutics research project investigating requirements for a secondary malignancies MDR-LT. EsPRit was refined based on the lessons learned in this case study.

## Methods

### Long-term Medical Data Registries (MDR-LT)

As defined by Arts et al. [9] in 2002, a Medical Data Registry (MDR) is: “a systematic collection of a clearly defined set of health and demographic data for patients with specific health characteristics, held in a central database for a predefined purpose.” Although this definition accounts for the basic purpose of registries it does not reflect recent technological developments such as distributed storage of medical data as, for example, proposed by state of the art electronic health record concepts [10]. A more abstract definition is introduced by Drolet et al., who define five essential characteristics for each MDR [11]:

- (M)ergeable data: Data format to aggregate all patient data for research and patient care
- (D)ataset standardized: Storage of the same data elements for all patients
- (R)ules for data collection: Definition of how to collect data
- (O)bservations associated over time: Ability for storage of longitudinal data
- (K)nowledge of outcomes: Support for Follow-ups

In addition to these five characteristics Drolet et al. postulated that a kind of the inclusion principle for patients should be taken into considerations for a medical data registry (DZ: chronic disease; TH: acute/interventional).

Accordingly, long-term Medical Data Registries (MDR-LT) have to be understood as a special subset of MDR dedicated to supporting long-term investigations. These MDR-LT have very long follow-up and data-collection periods, typically exceeding 20 years.

### The case study setting

Within the field of radiotherapy, major advances have been made throughout the last years in treating patients who suffer from various forms of cancer. Many of these new therapies (e.g. volumetric-modulated arc therapy (VMAT) and intensity-modulated radiation therapy (IMRT)) have one thing in common compared to classical therapies: increased body volume exposure to low dose of radiation. Several studies showed that the development of secondary malignancies correlates with the intensity and distribution of radiation [12–14]. It is - for now - unclear how these recently developed methods in radiotherapy affect the development of secondary malignancies. Hence, a long-term follow-up of patients is critical in the study of potential effects and their confounders.

SEMPER (Secondary Malignoma - Prospective Evaluation of the radiotherapeutics dose distribution as the cause for induction) [15] was a project part of the COMET Center ONCOTYROL,^1^ which was funded by the Austrian Federal Ministries BMVIT/BMWFJ (via FFG) and the Tiroler Zukunftsstiftung/Standortagentur Tirol (SAT). One of the central objectives of SEMPER was to analyze requirements for a MDR-LT, which can be used to study long-term qualitative and quantitative outcomes of these kinds of radiotherapy. Especially long-term effects of an exposure of large body-volumes to low radiation dosage during treatment is an imperative aspect (e.g. increasing incidence of secondary malignancies). In order to reach this goal, a variety of clinical data of participating patients and controls must be collected in a consistent manner. Particularly the long-term perspective of the data collection process must be considered. Secondary malignancies may arise late after initial treatment, several years in the future. Therefore, relevant data must be collected throughout a long period of time (>20 years) as well as complete follow-ups for all included subjects. As patient mobility has increased and given the long observation period of these studies, the data collection process must reflect a multi-center approach that allows cross-site data integration.

### The “anatomy” of ethics committee proposals

The outline and content to be provided by an ethics committee proposal are mainly based on the Declaration of Helsinki and quality standard GCP. However, ethic committees adopt or extend these standards and guidelines to comply with local requirements, specific regulations and the applicable law. Usually, the committees provide templates to support researchers to create ethics proposals.

GCP defines several documents which have to be provided to (independent) ethics committees: “*trial protocol(s)/amendment(s), written informed consent form(s) and consent form updates that the investigator proposes for use in the trial, subject recruitment procedures (e.g. advertisements), written information to be provided to subjects, Investigator’s Brochure (IB), available safety information, information about payments and compensation available to subjects, the investigator is current curriculum vitae and/or other documentation evidencing qualification*s” [6]. The trial protocol has to contain exact information regarding methods of analysis and data elements to be collected in form of Case Report Forms (CRF).

The recommended structure of a research protocol is outlined by the World Health Organization (WHO) [16]. As with the GCP guidelines, the WHO guidelines require that all data elements, as well as the data analyzing methods, have to be specified prior to the commencing of the study. This neither accounts for a possible change/extension in methods nor for the analysis or for changes in data elements over time.

### Elaboration of EsPRit

MDR-LT projects widely use up-to-date software tools [17]. These tools are usually developed following defined software engineering paradigms such as the waterfall model [18], eXtreme Programming (XP) [19], Rational Unified Process (RUP) [20], SCRUM [21] or the Agile Unified Process (AUP) [22]. The base and ongoing input of any of these software engineering paradigms are requirements that have to be provided by the project’s stakeholders or elaborated on together with said stakeholders either at the begin or during implementation. Although the concrete requirements can vary depending on the respective software project, the collection and validation of requirements can be standardized to some extent. The concept of Requirements Engineering (RE) [23, 24] outlines the processes and tasks to manage requirements on a general level (define, document, maintain, validate). The following common sub processes in RE can be identified:

- Requirements inception and elicitation: Collect an initial set of requirements and ideas for the project to implement
- Requirements identification: Identify requirements based on the project goal and the stakeholders involved
- Requirements analysis and negotiation: Harmonize the various requirements of involved stakeholders
- Requirements specification: Prepare requirements e.g. software development and document the requirements
- System modeling: Model requirements using appropriate modeling languages such as the Unified Modeling Language (UML) [25] version 2 or the Business Process Model and Notation version 2 (BPMN2) [26]
- Requirements validation: Validation of the collected requirements
- Requirements management: Keep track of changed requirements and update documentation

These sub processes are reflected in the EsPRit approach. As depicted before, MDR-LT have to deal with certain uncertainty concerning necessary data elements as well as the relevant research questions and can be subject to various changes during their lifetime. Thus, the base EsPRit model was enriched with components of the SPIRIT framework [8].

SPIRIT is a conceptual best-practice framework for the systematic planning of intelligent reuse of integrated clinical routine data. It can be applied to overcome the gap in situations where reuse of data was not intended. This often leads to the rarity of explicit research questions for secondary use initiatives of clinical data in the planning phase of such data reuse solutions. In many cases, additional and more definite research questions emerge once a critical mass of data has been accumulated and first investigations lead to a crystallization of plausible research hypotheses. SPIRIT suggests an iterative modus operandi to leave scope for development and evolution in such situations, which was adapted for EsPRit.

General MDR-LT requirements collected in a systematic literature review [27] prior to this work were then used to infer and align groups of tasks (building blocks) that have to be carried out to formulate a proper ethics proposal. According to the general requirements, a set of appropriate methods was defined for each building block. The resulting EsPRit model proposes an iterative workflow along the RE sub-processes and defines generic building blocks for an ethics proposal.

To test the feasibility and practicability of EsPRits an ethics committee proposal was compiled for SEMPER. The lessons learned during this case study were then used to further refine the EsPRit approach. A crucial insight that was gained was, for example, that consensus on an initial data set for the MDR-LT was very hard to find. Therefore, the central EsPRit process of electronic case report forms (eCRF) design (see below) was adapted to better support an iterative data set definition starting with a minimal core MDR dataset.

To represent the EsPRit approach (processes, building blocks, methods, results) BPMN2 process modeling is used for specifying business processes. The concept of Swim Lanes was adopted to model the main building blocks; these lanes consist of a pool and a lane. Here, the pool represents the main activity of the block, whereas the lanes summarize tasks to retrieve information. For better readability, the names given to these tasks indicate the information source. Each block produces an outcome (data object). The methods to retrieve and process information of each block are outlined as annotations.

## Results

The following section introduces the EsPRit approach (Ethics Proposals for Long Term Medical Data Registries). Since the final content and structure of long-term MDR-LT is not necessarily clear from the beginning, an approach not solely focusing on the data and structural perspective is needed.

Regulating and evaluating the data and structural perspective of an MDR-LT may be one way of safeguarding study quality and patient well-being but is not limited to this. In the field of medicinal products -and especially in the sub-domain of medicinal software products- two ways are possible to obtain certification. In the European Union a product can be certified by a clinical study or, as is more commonly seen in the software sub-domain, by regularly evaluating the existing literature (risk management) together with the application of procedural standards regarding the conceptualization, implementation and continuous evaluation of the product.

Although a complete introduction of the approach would be too extensive, it foresees the application of general process standardization such as the ISO 9000 series and the implementation of a risk management e.g. ISO 14971. The procedural approach in medicinal product certification therefore combines two important aspects: it assures product safety and allows for the inclusion of new or changed knowledge over time. Thus, finally yielding better product quality while assuring no risk of harm regarding all people involved (e.g. patient, healthcare professional).

EsPRit follows a comparable procedural approach, which guarantees a unified and traceable execution of predefined actions in the course of the design of an MDR-LT. It defines five major groups of possible activities, information sources, methods and results (building blocks) as well as their relation.

In order to practically apply EsPRit, it is necessary to create a document that describes the methods used for each activity (e.g. systematic literature review, qualitative expert interview, focus groups, document analysis, etc.) including their parameters (e.g. selected literature databases, exact search terms, applied guidelines such as “Preferred Reporting Items for Systematic Reviews and Meta-Analyses” (PRISMA), inclusion and exclusion criteria for experts, etc.). The resulting document may then be used as part of the ethics proposal, replacing or complementing the research protocol. The compiled result is forwarded to the ethics committee for decision making.

### EsPRit: building blocks and methods

**Fig. 1:**
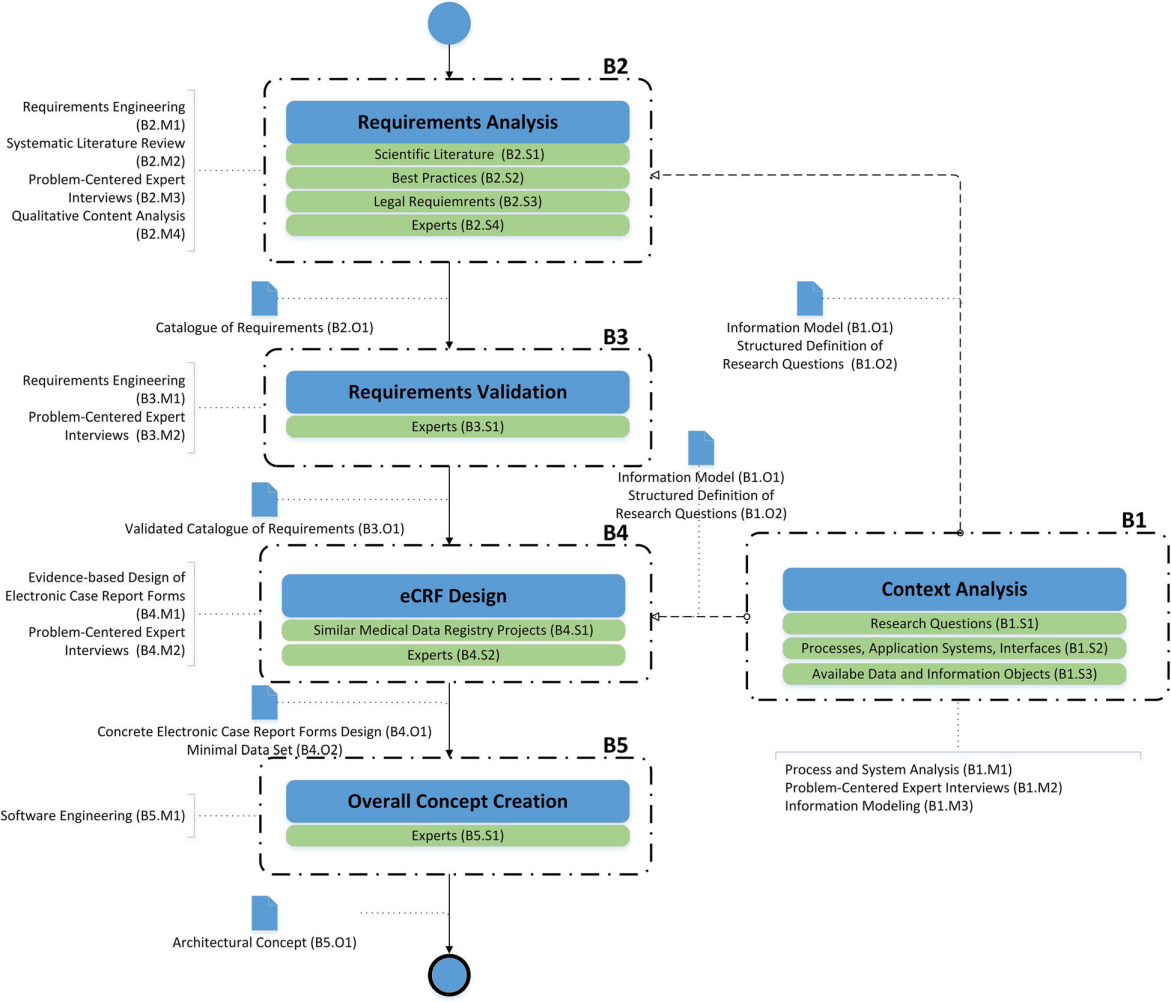
Overview on Building Blocks and attached Methods of EsPRit

Figure 1 outlines the five building blocks (surrounded by dashed rectangles). Each block contains a main activity (blue rectangles). Green rectangles describe assigned tasks that have to be carried out in order to collect necessary information. For a better readability the task’s names refer to the data sources for this information (S). The methods (M) that can be used to process or collect the information are presented as annotations to the block. The outcomes (O) of the blocks are represented as data objects (blue document icons). For an initial setup in creating an ethics committee proposal the order as outlined in the process model has to be followed. Special attention has to be paid to the block “Context Analysis” (B1) as it derives information of the “Requirements Analysis” (B2) as well as for the “eCRF Design” (B4) and must be carried out in synchronization with these blocks.

The following sections describe each building block (B1 – B5) with its main activity, the information sources, methods and outcome.

#### B1 - Context analysis

The activities and tasks in this block discover context-based circumstances for the MDR-LT. This includes documenting and modeling the current situation within the area of interest (e.g. relevant, information and documentation systems, diagnostic and therapeutic processes, available data elements, interfaces of these systems). In addition, the research question(s) for the long-term study have to be defined. The outcome of this step is used to prepare the design of Case Report Forms (B4) and to complement the input for the Requirements Analysis (B2). A summary of information sources, methods and the outcome can be found in Table 1.

**Table 1:** Information sources, methods and outcome of B1 - Context analysis

Information source (S)	Methods category (M)	Outcome (O)	Possible methodical approaches
Research Questions (B1.S1)	Problem-Centered Expert Interview (B1.M2)	Structured definition of the research question (B1.O2)	SPIRIT [8]
Processes, Application Systems, Interfaces (B1.S2)	Process and System Analysis (B1.M1)	Specific model of the processes, application systems and interfaces with respect to the research question (B1.O1)	Three-Level Graph-Based Model for the Management of Hospital Information Systems (3LGM^2^) [39] SPIRIT
Available data and information objects (B1.S3)	Information Modeling (B1.M3)	Structured description of data elements and information objects to answer the research question (B1.O1)	3LGM^2^ SPIRIT

##### B1.S1 - Research questions

The research question implicitly defines relevant processes and information systems as well as the necessary information objects in order to implement the MDR-LT. The proper definition and analysis of a research question including potential future clinical questions to be answered is essential to guarantee the alignment of data contained in the MDR-LT with latter reporting.

##### B1.S2 - Processes, application systems and interfaces

The analysis involves diagnostic and therapeutic processes that produce or process data, clinical application systems, application system interfaces and their documentation. These information sources allow for an insight into how relevant data is collected and can be extracted as well as how these data can be integrated in the MDR-LT (e.g. by connecting to web service interfaces of the application systems). To ensure high quality data contained in an MDR-LT it is important to carefully investigate the processes of data collection and processing in order to identify and avoid potential errors at the stage of conceptualization.

##### B1.S3 - Available data and information objects

The data objects to be discovered in this block together with the outcome of B1.S2 form the initial pool from which relevant data objects, with respect to the research question, may be selected to be incorporated into the MDR-LT. The analysis of available information and data objects allows for the identification of currently missing data needed to answer the research question.

##### B1.M1 – Process and system analysis

The current data collection process and involved application systems provide insights concerning which and how data is collected and how this data can be incorporated. To obtain all necessary information with respect to the research question the first proposed approach *(Top-Down)* as outlined in the SPIRIT framework [8] may be used:

- *Top-Down:* Analysis of the research question(s), which shall be answered through the implementation of the MDR-LT. The research question should be defined in a structured way. To outline the data elements based on the research question, one can conduct problem-centered expert interviews [28] with medical domain experts within the study.

To analyze the discovered information systems, the second proposed approach *(In-Between)* of the SPIRIT framework may be used:

- *In-Between:* Analysis of tasks and the fields of activity of the areas of interest. Additional, analysis of diagnostic and therapeutic processes including produced medical documentation and used information systems and their interfaces.

To collect said data the third proposed approach *(Bottom-up)* of the SPIRIT framework may be used:

- *Bottom-up:* Analysis of available data and information objects.

The collected data objects should be described in a structured way and are added to the information model as a main outcome of this block.

##### B1.M2 – Problem centered expert interviews

Domain experts provide knowledge about existing and needed data elements and data collection processes, regarding how to achieve the goal of answering the research question. To formalize the process of collecting this knowledge, problem-centered expert interviews should be conducted. The transcript of the interviews can be analyzed by using methods of qualitative content analysis.

##### B1.M3 – Information modeling

The discovered information including application systems, interfaces, diagnostic and therapeutic processes and interdependencies provided by the information source of this block shall be presented in a structured way. To model this information, a suitable modeling technique like the 3LGM^2^ can be used. This meta-model provides three layers (functional, logical, and physical) to add the discovered elements like the application systems, etc.

##### B1.O1 – Information model

The information model contains all processes and information sources, which produce or process data in the field of interest. The identified data elements needed to answer the research question should be listed in a structured way. For each data element the title, the source (available information system used in routine car; needs to be collected prospectively, etc.), the coding standard (e.g. ICD-10, SNOMED CT, etc.), the data type (number, string, etc.) and the storage location (e.g. local, external partner, governmental contact point, etc.) should be described. The elaborated model provides an important input for subsequent blocks and shall be presented in a standardized way (e.g. 3LGM^2^).

##### B1.O2 - Structured definition of the research questions

The research question should be presented in a structured way. The structured definition of the research questions is used to infer the required data elements and limits the functionality of the MDR-LT to a set needed to answer the research question. The inferred data elements and the functionality restrictions are used as inputs for subsequent blocks.

#### B2 - Requirements analysis

Within this block, the requirements for the concrete MDR-LT implementation are collected. The information model and the structured description of the research question elaborated in “Context Analysis” serve as the basis. The collected requirements reflect the functionality of the specific MDR-LT project. Additionally, requirements are collected using several information sources such as scientific literature, best practices, legal demands and domain experts. A summary of information sources, methods and the outcome can be found in Table 2.

**Table 2:** Information sources, methods and outcome of B2 - Requirements analysis

Information source (S)	Methods category (M)	Outcome (O)	Possible methodical approaches
Scientific Literature (B2.S1)	Systematic Literature Review (B2.M2)	Catalogue of Requirements (B2.O1)	Qualitative Content Analysis [40] PRISMA [41]
Best Practices (B2.S2)	Systematic Literature Review (B2.M2)	Catalogue of Requirements (B2.O1)	Qualitative Content Analysis PRISMA
Legal Requirements (B2.S3)	Systematic Literature Review (B2.M2)	Catalogue of Requirements (B2.O1)	Qualitative Content Analysis PRISMA
Experts (B2.S4)	Requirements Engineering (B2.M1) Problem-centered Expert Interviews (B2.M3)	Catalogue of Requirements (B2.O1)	UML [25] Qualitative Content Analysis

##### B2.S1 – Scientific literature

To retrieve the necessary requirements regarding the specific MDR-LT project, existing and comparable projects published in scientific literature should be screened. An example of general requirements regarding MDRs is published by the European Clinical Research Infrastructure Network (ECRIN) [29]. ECRIN provides a list of standard requirements for managing of IT in trial units. The revised list contains three main categories: IT Standards (e.g. Management of Servers, Physical Security, etc.), Data Management Standards (Data Entry and Processing, Data Quality Checks, etc.) and General Standards (Transferring Data, Long Term Data Storage, etc.). The requirements outlined in ECRIN can be used and/or extended to meet the specific aim of the MDR-LT to be built.

##### B2.S2 – Best practices

Best practice approaches summarize experiences from already implemented MDR projects. Considering this, information and inferred requirements regarding the specific MDR-LT project prevents possible pitfalls during implementation.

##### B2.S3 – Legal requirements

Additionally, national legal requirements must be taken into account (e.g. requirements regarding data privacy, data protection, etc.). The MDR-LT must adhere to those governmental rules. Usually requirements inferred from suitable laws refine the already collected requirements from scientific literature and best practice approaches.

##### B2.S4 – Experts

Medical domain experts outline the requirements from their perspective, envisioning the special setting where the MDR-LT will be applied to. Taking these requirements into account can increase the user acceptance which serves as a key factor for a successful study project [30].

##### B2.M1 – Requirements engineering

This type of analysis focuses on requirements elicitation, requirements analysis, requirements specification and requirements validation. The collected requirements should be described and documented. To model the Use Cases of each requirement, UML Use Case diagrams can be used.

##### B2.M2 – Systematic literature review

The method of a systematic literature review can be used to screen scientific publications of MDR projects, product description of commercial systems, best practices or legal demands. To formalize the actions within this step the PRISMA statement should be considered. By following this process, traceability of the requirements identification and collection can be ensured.

##### B2.M3 – Problem-centered expert interviews

To formalize the process of collecting requirements from domain experts, problem-centered expert interview should be used. Again, following such a method increases the traceability of the requirements elicitation phase. The transcript of those interviews can be processed e.g. by qualitative content analysis methods.

##### B2.O1 – Catalogue of requirements

The outcome of this block is a catalogue of functional and nonfunctional requirements including its description. The needed functionality of the MDR-LT is outlined based on the requirements. The requirements should be presented in a structured way. The catalogue is passed to the Requirements Validation block.

##### B3 - Requirements validation

Within this block of activities, the initially collected requirements are refined and improved. A first validation is done within the requirements analysis phase B2. Nevertheless, this step extends the validation carried out before. The identified requirements are validated to align and tightly couple the MDR-LT to the research question, identify possible pitfalls or remove unnecessary requirements. The review can be formalized by formulating requirements as user stories. A summary of information sources, methods and outcome can be found in Table 3.

**Table 3:** Information sources, methods and outcome of B3 - Requirements validation

Information source (S)	Methods category (M)	Outcome (O)	Possible methodical approaches
Experts (B3.S1)	Requirements Engineering (B3.M1) Problem-Centered Expert Interview (B3.M2)	Validated Catalogue of Requirements (B3.O1)	Infer and Evaluate User Stories

##### B3.S1 - Experts

The main information sources for this block are domain experts. The knowledge of those should be used to validate the inferred requirements. Collected requirements are presented and discussed until a consensus is reached concerning whether these should be included in the catalogue of requirements or not.

##### B3.M1 – Requirements engineering

The validation of the collected requirements is carried out in more depth and enriched with information provided by domain experts. The collected requirements can be formulated as user stories. The concept of user stories was first specified in the software engineering paradigm of XP [19]. A user story is a description expressing the requirements from the users perspective (e.g. “As a user I want to login so that I can enter patient specific health data”). Such statements consist of three parts (see example): *{user}* - as the role which wants to perform an action, *{want to login}* - as the action and *{I can enter patient specific health data}* - as the perceived benefit. The benefit should be presented to domain experts and stakeholders in order to make an informed decision regarding whether the requirement is necessary to meet the study goal. Requirements that do not pass this validation should be removed from the catalogue of requirements. A similar approach of validating the requirements can be found within the KoRegIT project [31].

##### B3.M2 – Problem-centered expert interviews

Domain experts should review the collected requirements. The initial catalogue is revised according to the feedback of those experts. To formalize this process, problem-centered expert interviews should be conducted. The initial catalogue of requirements and the inferred user stories are presented and discussed with these experts. A decision is made on whether to include or exclude the focused requirement based on said discussion.

##### B3.O1 – Validated catalogue of requirements

The outcome of this block is an extended (or downscaled), adjusted and validated catalogue of requirements. The catalogue forms the functional basis of the MDR-LT to be built and is the major input for B5 to create the overall architectural concept.

#### B4 - eCRF design

The core of each MDR-LT is formed by eCRFs. These are specialized documents that outline the data elements to be collected, which are necessary to answer the research question. Data should be collected unambiguously and in sufficient detail but should avoid redundancy or elements that are too detailed. A guide on how to design eCRFs can be found in [32].

Within this block, specialized documents should be created based on the outcome of B1, which provides a structured definition of the research question (B1.O1) and the already available data pool (B1.O2). Data elements from the pool of available data are selected to design the eCRFs. If needed, additional data elements are defined, which then should be collected in a prospective manner.

Additionally, a minimal data set for the MDR-LT should be created. This set contains data elements that mustn’t change over time, as to ensure that basic analysis can be carried out at any time during the lifecycle of the MDR-LT.

A summary of information sources, methods and the outcome can be found in Table 4.

**Table 4:** Information sources, methods and outcome of B4 – eCRF design validation

Information source (S)	Methods category (M)	Outcome (O)	Possible methodical approaches
Similar Medical Data Registry (MDR) Projects (B4.S1)	Evidence-based Design of Electronic Case Report Forms (eCRFs) (B4.M1)	Concrete eCRF Design (B4.O1)	Qualitative Content Analysis [40]
Experts (B4.S2)	Problem-Centered Expert Interview (B4.M2)	Minimal Data Set (B4.O2)	Qualitative Content Analysis

##### B4.S1 – Similar MDR projects

Similar existing studies within the area of interest should be taken into account in order to create meaningful and useful eCRFs to answer the research questions. These sources provide suggestions regarding what data elements are needed to answer the research question.

##### B4.S2 – Experts

The knowledge and experience of medical experts is used to identify the data elements essential in answering the research question.

##### B4.M1 – Evidence-based eCRFs design

As mentioned previously, information sources of particular importance are MDR-LT projects that are similar or related. To reduce the effort in identifying needed data elements, such studies should be evaluated to identify and correlate the data elements. An additional benefit of this approach is the comparability and validation possibilities concerning the outcome of the study.

##### B4.M2 – Problem-centered expert interviews

To formalize the process of collecting requirements from domain experts, problem-centered expert interviews should be conducted. Adhering to this method ensures traceability and verifiability of the data elements that are needed to answer the research question.

##### B4.O1 – Concrete eCRF design

One outcome of this block is a description of a concrete eCRFs design suitable for answering the research question. The items to be collected can be grouped in different levels (form, group, section, item) in order to standardize the description [33]. Additionally, for each data item a coding standard (e.g. SNOMED CT,^2^ LOINC,^3^ ATC,^4^ ICD,^5^ etc.) and a suitable code should be specified. Furthermore, the appropriate documents needed for patients to provide informed consent can be developed based on the specified eCRF design.

##### B4.O2 – Minimal data set (MDS)

A minimal data set is the minimum of data elements needed to answer the initial research questions. GCP and the study protocol definition by WHO outline that the case report form has to be defined prior to the implementation of the study. In long-term data collection processes the needed data elements are subject to changes. To overcome the outlined contradiction a MDS should be defined for a MDR-LT. The MDS mustn’t change over time in order to guarantee that initially designed data analysis methods can be executed at any time of the study’s lifecycle. Examples for MDS in medical research can be found in several MDR projects [34–36].

#### B5 - Overall concept creation

Within this block all prior information and knowledge produced are merged together to create an overall concept for the MDR-LT project. The elaborated architecture serves as the foundation on which the MDR-LT is to be implemented. Methods from software engineering are used to compile the architecture of the MDR-LT. A summary of information sources, methods and outcome can be found in Table 5.

**Table 5:** Information sources, methods and outcome of B5 – Overall concept creation

Information source (S)	Methods category (M)	Outcome (O)	Possible methodical approaches
Experts (B5.S1)	Software Engineering (B5.M1)	Architectural Concept (B5.O1)	Agile Unified Process (AUP) [22] Entity-Relationship Models [37]

##### B5.S1 - Experts

The main information source for this block comprises experts mainly selected from the domain of software engineering. The knowledge of these experts is used to incorporate the derived requirements and eCRFs that are then merged into a feasible architectural software concept.

##### B5.M1 - Software engineering

Software Engineering as a method focuses on all activities to develop, test and maintain software. Additionally, the modeling of data structures and the operation of software products are covered.

UML can be used to model the software architecture and provides several diagrams that describe the structure and behavior of software systems. The data objects, which should be stored e.g.in relational databases, can be modeled by using the Entity-Relationship Model [37]. The lifecycle management of MDR-LT software systems such as maintenance or deployment can be described using the AUP software engineering paradigm [22].

##### B5.O1 – Architectural concept

As an outcome of this block, an architectural concept of the MDR-LT that is to be implemented is provided. With this concept, the ethics committee proposal can be extended in order to provide all the needed information for the committee to make a well-founded decision on whether the projects meets the required ethics standards.

### Lifecycle management

At compile time of the ethics committee application, or even after the document for submission had been created, new requirements (e.g. regarding data elements for the MDR-LT) may be detected. Additionally, as outlined before, once completed, the ethics committee application may be subject to change, e.g. due to new data elements that needed to be. To reflect these possibilities, it is proposed that only the affected (e.g. B4 – eCRF Design) and the subsequent blocks (B5 – Overall Concept Creation) must undergo reconsideration, since other blocks are left unaffected. The lifecycle management should be carried out in an iterative manner as proposed by software engineering approaches such as AUP.

If opinions of medical experts differ on fundamental issues like study goals (B1) or necessary data sets to answer the research question (B4) a consensus has to be reached. If a consensus cannot be reached the designers of the MDR-LT have to decide whether to proceed preparing the application or revise the initial goal of the study. Any concerns regarding the feasibility have to be documented in a comprehensible way.

The study designers describe the process on how to e.g. discover MDR-LT requirements to meet the goal of the study. The process should adhere to well proven and documented methods. If an ethics committee decision requires changes to the described process the comments have to be analyzed and if feasible incorporated into the application. Since the process of compiling the application is iterative not all building blocks have to be reconsidered.

At any point in time during the life cycle of a MDR-LT an ethics committee proposal compiled using EsPRit is a fundamental part of the documentation of the registry. If, for example data collection methods change over time the adaption has to be reflected in the ethics committee proposal. Thus, a new decision covering the changes has to be made by the committee. Therefore, at any point in time and for each data item it is well documented how the data was collected and how the data was interpreted.

## Discussion

### EsPRit in a nutshell

The EsPRit approach defines building blocks, activities, methods and a process to systematically design MDR-LT in a goal-oriented, standardized and reproducible way. Thus, it can be used to compile the necessary ethics committee proposals for a planned MDR-LT by defining and describing the concrete activities, information sources and methods within each building block. This process-oriented approach follows the idea put forth in medicinal product certification. In contrast to a content-based approach, the outcome of each block must not be defined in advance. A system design adhering to EsPRit will lead to an MDR-LT that will be in accordance to the high ethical standards required by medical research. Thus, a certifying body, e.g. an ethics committee is able to make an informed decision already in the planning phase of the MDR-LT.

### Limitations

However, the decision on whether to permit or decline a research project on the grounds of ethical reasoning is highly dependent on the local implementation of ethical standards. The committee has to support the shift from a content-based approach to one that is process-oriented, ensuring that the research project complies with the implemented ethical standards. Additionally, the commitment to adhere to the defined process outlined by EsPRit is an absolute prerequisite on behalf of the researchers involved in implementing a MDR-LT. Especially a change management process must be implemented in order to reflect the evolution of the MDR-LT during its life cycle. These changes have to be communicated to the ethical committee in an appropriate way. The committee must then revise those changes. In this case, it is not necessary to examine every part of the initial ethical committee application; only changed parts (e.g. added data items in an eCRF) have to be reconsidered. Ethical committees will have to adapt to this new shift by providing, for example, new guidelines that reflect this approach as well as the local requirements.

Compiling an ethics proposal as proposed by EsPRit may add overhead to the initial planning phase of an MDR-LT. Nevertheless, the elaborated building blocks for the ethics proposal can be reused as an initial draft description for the concrete MDR-LT implementation. The inferred overall concept forms the basis of, for example, an implementation request to a software development company building the concrete MDR-LT. The software implementation process is independent of a specific implementation process model as long as the software fulfills the elaborated requirements.

During the initial compilation of the ethics committee proposal in the SEMPER case study, the eCRF was subject of change due to the research question’s vague specification. Additional data elements had to be added during the elaboration of the MDR-LT concept. However, since the Requirements Analysis (B2) and the Requirements Validation (B3) were not affected (as adding new data elements does not affect requirements e.g. regarding data storage, etc.), these blocks had not to be reconsidered. Within the Overall Concept Creation (B5) the outlined agile processes defined by software development allowed for the integration of these new data elements very easily into the final MDR-LT concept. Within SEMPER, utilizing EsPRit as well as the inferred eCRF, an excerpt of the created proposal was used to compile a traditional ethics committee proposal. This decision was made in conjunction with the SEMPER team and the ethics committee in order to ensure on-time implementation of the MDR-LT, since implementing the new approach of compiling ethics committee applications using EsPRit requires further evaluation regarding its feasibility and applicability in medical research routine.

### EsPRit in relation to other approaches

The switch to a process-oriented approach in creating medical committee applications provides a new way of ensuring high ethical standards in medical research. As far as we know, this kind of procedure has not been proposed yet. In conjunction with existing standardizing efforts in the field of data management for clinical trials and MDRs, the complexity of creating an ethics committee proposal using the proposed process-oriented EsPRit approach can be reduced. ECRIN is a standard which provides 115 IT requirements, 107 Data Management requirements and 13 other requirements to provide GCP-compliant data management for clinical trials [1]. Using these requirements as a MDR-LT following the EsPRit approach will reduce the efforts to elaborate the building blocks B2 and B3. Of course, additional requirements reflecting the special setting for which the MDR-LT is to be developed for will have to be considered as well.

A crucial part of a MDR-LT is the integration of existing routine data. Projects mainly focusing on reusing existing routine data for scientific purposes are, for example, “Electronic Health Records for Clinical Research” (EHR4CR)^6^ or “Retrieve from EHR Useful clinical data for Secondary Exploitation” (RE-USE) [38]. Both projects focus, while using standard-based tools and services (e.g. based on Integrating the Healthcare Enterprise (IHE) profiles, etc.), to extract data from electronic health records and provide these data sets to clinical research in a meaningful und useful. Additionally, single-source data entry, collection and cross-system data reuse shall be supported. Especially the RE-USE project focuses on technical solutions to enable semantic integration of different data sets. When using these data sets for clinical research, ethical aspects have to be taken into account as well. Approaches developed within these projects can be integrated within EsPRit to provide technical requirements of the data management in a MDR-LT. The process-oriented approach allows the addition and refinement of the technical methods during the development and runtime of the MDR-LT without the need to rewrite the complete ethics committee proposal.

The added value of EsPRit for MDR-LT projects results in reliable planning and thus, when adhering to this process, an increased compliance to ethical guidelines over time.

### Open questions and outlook

Legal aspects such as patient data privacy in long-term studies or the reporting of quality data sets for national health planning tasks must be taken into account in order to use the EsPRit approach in setting up MDR-LTs. It must be reviewed whether the high ethical standards in clinical research are sufficiently reflected. Established ethics committees have to decide whether they accept such proposals or whether additional information must be provided to ensure such things as patient rights. Especially due to changes of the MDR-LT over time, such as in terms of data items to collect, ethic committee proposals created based on the EsPRit approach can reduce the time to provide an adapted application for the committee. Thus far it remains unclear whether those adapted versions are sufficient in ensuring high ethical standards in clinical research. For evaluation of the proposed approach, this long-term perspective must be strongly taken into account.

The initial compilation of an ethics committee proposal using the EsPRit approach may consume additional time and effort in comparison to the traditional manner. Nevertheless, in the long-term it makes sense to spend these additional efforts since such a proposal can support the lifecycle management of MDR-LTs and can be reused for new MDR-LT projects. Only requirements dedicated to the new setting of the MDR-LT being developed would have to be added in such a case. Adapting the building blocks B1 and B4, which are mainly used to reflect the clinical research questions, should be sufficient for most projects.

Nevertheless, within the SEMPER research project, a process-oriented approach was used to create a basic MDR-LT construct, covering the long-term perspective of data collection in order to answer a specific research question, as well as providing a structure to better represent a volatile field in medical research. The result showed that the inferred building blocks and the process-oriented approach of EsPRit provide a good structure using a process-oriented platform in inferring ethical committee applications.

## Conclusion

EsPRit provides an opportunity to streamline the compilation of ethics committee proposals. EsPRit helps to create valid and meaningful ethics committee proposals at early stages of long-term projects, at a time when information is often scarce. It minimizes the risk of rejection by following well-established methods, thus increasing patient safety and confidentiality. As long as the study organizers guarantee compliance to the EsPRit approach, an informed and well-founded decision can be made by the ethics committee as to whether accept or decline the implementation of the desired MDR-LT and proposed medical research project. The focus for this decision mustn’t be set on technical-ethical requirements but can remain focused on medical-ethical aspects.

## Abbreviations

3LGM^2^: Three-level graph-based model for the management of hospital information systems
BPMN2: Business Process Model and Notation version 2
CRF: Case Report Forms
eCRF: Electronic Case Report Forms
ECRIN: European Clinical Research Infrastructure Network
EHR4CR: Electronic Health Records for Clinical Research
EsPRit: EthicS Proposal RegIsTry
GCP: Good Clinical Practice
IMRT: Intensity-modulated radiation therapy
IT: Information Technology
MDR: Medical Data Registries
MDR-LT: Long-Term Medical Data Registries
MDS: Minimal data set
PRISMA: Preferred Reporting Items for Systematic Reviews and Meta-Analyses
RE: Requirements Engineering
RE-USE: Retrieve from EHR Useful clinical data for Secondary Exploitation
RUP: Rational Unified Process
SEMPER: SEcondary Malignoma - Prospective Evaluation of the radiotherapeutics dose distribution
SPIRIT: Systematic Planning of Intelligent Reuse of Integrated Clinical Routine Data
UML: Unified Modeling Language
VMAT: Volumetric-modulated arc therapy
WHO: World Health Organization
WMA: World Medical Association
XP: eXtreme Programming

## Footnotes

1
http://www.oncotyrol.at (accessed January 8, 2016)

2
https://www.nlm.nih.gov/healthit/snomedct/, accessed 02-02-2016

3
https://loinc.org/, accessed 02-02-2016

4
http://www.whocc.no/atc_ddd_index/, accessed 02-02-2016

5
http://www.who.int/classifications/icd/en/, accessed 02-02-2016

6
http://www.ehr4cr.eu/, (accessed July 2, 2016)
